# Identifying a Disc-shaped Foreign Body

**DOI:** 10.5811/cpcem.2019.1.41829

**Published:** 2019-03-05

**Authors:** Michael Lee, Michael George, Kate E. Dorney

**Affiliations:** * Boston Children’s Hospital, Division of Emergency Medicine, Boston, Massachusetts; † Boston Children’s Hospital, Division of Radiology, Boston, Massachusetts

## Abstract

A four-year-old girl presented to the emergency department vomiting after a foreign body ingestion. An anteroposterior plain radiograph demonstrated a disc-shaped foreign body. Ordinarily, a plain radiograph cannot conclusively identify an object as a coin rather than a button battery that requires emergent removal. However, this high-voltage radiograph, windowed to increase contrast, showed the visible face of George Washington to confirm the diagnosis of an ingested quarter.

## CASE PRESENTATION

**Image 1:**
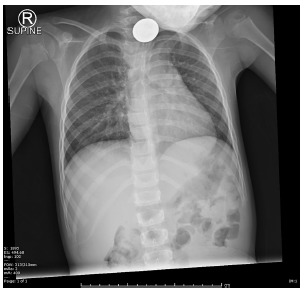
Anteroposterior plain radiograph of the chest, demonstrating disc-shaped foreign body. The en face view suggests but does not prove likely esophageal location.

**Image 2:**
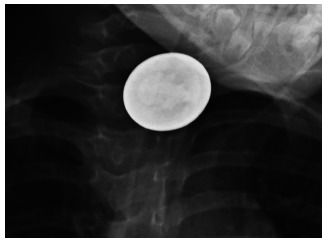
Plain radiograph of the chest, windowed to increase contrast. The visible face of George Washington identifies this object as a quarter rather than a button battery.

**Image 3:**
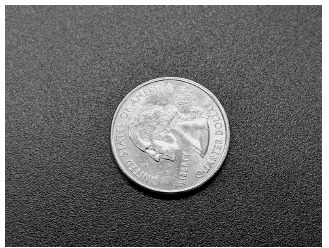
Photograph of a quarter, in a similar orientation to the quarter in Image 2.

A four-year-old girl presented to the emergency department with several episodes of vomiting; on questioning, she stated that she had swallowed something at daycare. Her parents were unsure whether she had access to button batteries. She had no respiratory distress on physical exam. The hospital obtained a single plain radiograph, an anteroposterior (AP) view of the chest (Image 1), and referred her to our tertiary center. The patient had no lateral film, but on high-contrast windowing of the film, which had been taken at a peak kilovoltage (kVp) of 100, the visible face of George Washington identified the object as a quarter (Images 2 and 3). The quarter was removed endoscopically without complication.

## CASE DISCUSSION

In a disc-shaped foreign body ingestion, identifying the foreign body is critical but can be challenging. Esophageal button batteries must be removed emergently, given the high risk of injury, while an esophageal coin can be removed urgently unless the patient cannot manage her secretions.^1^ On radiographs, a double halo on the AP view or a step-off seen on a lateral view suggests that a round object is likely to be a button battery.^1^ Although the location cannot be definitively determined without a lateral film, an object seen en face in the AP view is more likely to be esophageal than tracheal.^2^,^3^ Additionally, our patient’s symptoms – vomiting and dysphagia without respiratory distress – suggested an esophageal location.

In our patient, the unusual visibility of George Washington’s face was possible because the image was acquired at 100 kVp, at the higher end of the dose range for patients of this age and size. While somewhat reducing subject contrast, this voltage allowed the beam to better penetrate metal and therefore enhanced the clarity of the quarter while delivering a lower radiation dose to the patient.^4^

**CPC-EM Capsule**

What do we already know about this clinical entity?

*When a disc-shaped foreign body is seen on a plain radiograph, differentiating between coins and button batteries is critical but can be challenging*.

What is the major impact of the image(s)?

*The attached radiograph, taken at a peak kilovoltage (kVp) of 100, demonstrated the visible face of George Washington, identifying the object as a quarter*.

How might this improve emergency medicine practice?

*kVp doses at the high end of the range for a patient’s age and weight and high-contrast windowing, may help identify metallic foreign bodies*.
